# A tool to evaluate physiotherapy clinical education in South Africa

**DOI:** 10.4102/sajp.v78i1.1759

**Published:** 2022-08-31

**Authors:** Vaneshveri Naidoo, Aimée V. Stewart, Morake E.D. Maleka

**Affiliations:** 1 Department of Physiotherapy, Faculty of Health Sciences, University of the Witwatersrand, Johannesburg, South Africa

**Keywords:** physiotherapy clinical education, programme evaluation, monitoring and evaluation, quality assurance, context, input, process, product (CIPP)

## Abstract

**Background:**

Physiotherapy clinical education is complex. The dynamic learning milieu is fluid and multidimensional, which contributes to the complexity of the clinical learning experience. Consequently, there are numerous factors which impact the clinical learning experience which cannot be measured objectively – a gap which led to the development of our study.

**Objectives:**

To develop, validate, and test the reliability of an assessment tool that evaluates the effectiveness and quality of physiotherapy clinical education programmes.

**Method:**

A mixed methods approach in three phases included physiotherapy academics, clinical educators, and clinicians throughout South Africa. Phase One was a qualitative study: focus group discussions determined items and domains of the tool. Phase Two established the content and construct validity of the tool, a scoring system and a name for the tool, using the Delphi method. In Phase Three, factor analysis reduced the number of items, and the feasibility and utility of the tool was determined cross-sectionally.

**Results:**

The Vaneshveri Naidoo Clinical Programme Evaluation Tool (VN-CPET) of 58 items and six domains was developed and found to be valid, reliable (α = 0.75) and useful. The six domains of VN-CPET include governance; academic processes; learning exposure; clinical orientation; clinical supervision and quality assurance and monitoring and evaluation.

**Conclusion:**

The Vaneshveri Naidoo Clinical Programme Evaluation Tool is a valid, reliable and standardised tool, that evaluates the quality and effectiveness of physiotherapy clinical education programmes.

**Clinical implications:**

This tool can objectively evaluate the quality and effectiveness of physiotherapy clinical education programmes in South Africa, and other health science education programmes, both locally and globally, with minor modification.

## Introduction

Currently, there are no tools to evaluate the quality and effectiveness of physiotherapy clinical education programmes. Several scholars have attempted to do this, but were unsuccessful because clinical education is complex, diverse, and multidimensional (Higgs 1993; Jette et al. 2014; McCallum et al. 2013; Stachura, Garven & Reed 2000; Strohschein, Hagler & May 2002). The curriculum review process by academic departments largely focusses on the theoretical component (learning objectives, activities and outcomes), while the structure and processes of clinical education are largely overlooked; yet clinical education is a core component of a physiotherapy undergraduate programme (Baldry Currens & Bithell 2000; Chetty et al. 2018; Delany & Bragge 2009; Higgs 1993; McCallum et al. 2013; Moghadam, Kashfe & Abdi 2017).

A standardised, valid, and reliable monitoring and evaluation tool will facilitate the summative and formative evaluation of physiotherapy clinical education programmes (Frye & Hemmer 2012; Persky, Joyner & Cox 2012; Stachura et al. 2000). The programme’s structure and processes will be analysed, not only the outcomes (Frye & Hemmer 2012; Owston 2008). This kind of inquiry will enable strategic quality assurance mechanisms to be incorporated, and high-quality clinical learning experiences for students are likely to be achieved. Therefore, it is imperative that the clinical education component of the curriculum is independently and objectively evaluated.

Evaluation tools provide the data required to track the implementation of processes, to determine the programme’s intended and unintended effects, and to establish the programme’s effectiveness. Moreover, valid, and reliable tools are needed to facilitate the complex evaluation process of physiotherapy clinical education, with its unique, multifaceted constructs. Thus, the purpose of our study was to develop and validate the reliability of an assessment tool that evaluates the effectiveness and quality of a physiotherapy undergraduate clinical education programme: A Programme Evaluation Tool.

## Methods and procedure

A three-phase exploratory, sequential design that included mixed methods was used to develop the tool (Ivankova, Creswell & Stick 2006). Data collection commenced country-wide following ethical clearance to conduct this study from the Human Research Ethics Committee of the University of the Witwatersrand (Wits) – (ethical clearance number: M210160), as well as ethical clearance and/or permission, and informed consent from seven out of eight academic departments and clinical departments: University of Cape Town (UCT); University of Stellenbosch (US); University of the Free state (UFS); University of Kwazulu-Natal (UKZN); University of the Western Cape (UWC); Sefako Makgatho Health Sciences University (SMU); Chris Hani Baragwaneth Academic Hospital (CHBAH); Steve Biko Academic Hospital (SBAH) and Helen Joseph Academic Hospital (HJ).

After the pilot study, and through purposive sampling, 81 key stakeholders involved in student training (academics, clinical managers and clinicians, including new graduates) participated in the focus group discussions (FGDs). Focus group discussions allowed the first author to collect national data, efficiently and cost-effectively (Jayasekara 2012). Fourteen FGDs, each with approximately eight participants, took place. Data saturation occurred after the eighth FGD. However, appointments for the FGDs had been made 1 year in advance, so 14 FGDs were conducted. The FGDs consisted of mixed groups of participants, or distinct groups, depending on participants’ availability. A broadly structured script with prompts was used. The recorded FGDs were transcribed verbatim, coded, categorised and themed inductively by the first author, using Tesch’s (1992) method of data analysis (Vuso & James 2017), and MaxQda, version 2018.2 (a qualitative data analysis tool). Thematic content analysis was also conducted by the co-authors and an independent qualitative expert. There was a high level of agreement on coding and themes, and disagreements were discussed. Prior to the thematic content analysis, the transcripts were checked by the first author for errors, and member-checks confirmed that true accounts of the FGD had been captured. Data and investigator triangulation ensured trustworthiness (Halcomb & Andrew 2005; Leech & Onwuegbuzie 2007), leading to Phase Two of our study. My bias as clinical co-ordinator (CC) was curbed by conducting FGDs, where a broad statement was used to elicit the data; as well as inductive coding, member checks and co-coders.

For the Delphi process, 79 FGD participants were invited to participate in Phase Two of our study to determine the face and content validity of the preliminary tool (two FGD participants from Phase One were excluded due to invalid email addresses). The preliminary tool of 131 questions was emailed to the participants, who were asked to decide on which items should remain in the tool. Two Delphi rounds were undertaken and an 80% agreement on items in each round was obtained to keep the item in the preliminary tool. There is no standard level of consensus, although 70% – 80% is usually adopted (Diamond et al. 2014; Maleka, Stewart & Hale 2017; Trevelyan & Robinson 2015).The third Delphi round confirmed a scoring system and a name for our tool. Following each Delphi round, the first author and the co-authors reviewed the comments and edited questions, as recommended by the participants, and as appropriate. Descriptive statistics (frequencies and percentages) were used to analyse the data.

Following the Delphi process, a Research Electronic Data Capture (REDCap) link (a secure web platform for building and managing online databases and surveys) (projectredcap.org) of the preliminary tool was emailed to 13 participants (heads of departments [HOD] and/or CC and/or undergraduate co-ordinators [UG]) of the eight academic physiotherapy departments in South Africa, to enable principal component factor analysis to reduce the number of items in the tool (Abdi & Williams 2010). The participants were requested to answer all the questions. The internal consistency of the items was determined using Cronbach’s alpha.

Phase Three of our study, a cross-sectional survey, was used to determine the construct validity of the tool. A REDcap link of the provisional tool and questions testing the feasibility and utility (Appendix 1) of the tool was purposively emailed to 35 participants nationally and internationally (HODs and/or CCs in universities in the countries listed in Appendix 2). The participants were requested to complete the 58 questions in the tool, and to answer the following open-end questions:

1. Does this tool evaluate what you consider to be important regarding clinical education?
2. What are the strengths of this tool?
3. Indicate the weaknesses of this tool.
4. Is this tool useful for your institution?

The data were tabulated, and descriptive statistics, frequencies, and percentages, were used to analyse the data. Principal component analysis was conducted using Stata (16.0).

### Ethical considerations

Our study was coducted under a strict code of ethics; the anonymity of all participants were maintained where possible; there was no identifying data on any of the data collection sheets and the data was handled under utmost confidentiality. The raw data was stored in a locked cupboard M210160. 20210204. A second ethics cleareance certificate was applied for as the first one (M140706 – 22/08/2014) expired.

## Results

### Phase one

**FIGURE 1:**
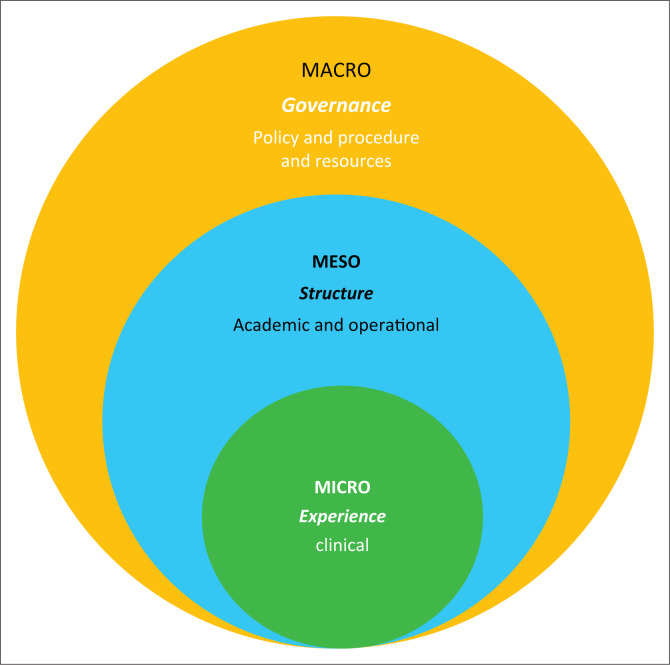
Themes: *Governance, structure and experience.*

The preliminary tool of 131 items which emerged following the FGDs, contained three key areas: *Governance, structure and experience;* the macro-, meso- and micro-components, respectively, as seen in Figure 1.

Table 1 provides an overview of the categories and subcategories under each theme.

**TABLE 1:** Summary of themes, categories and subcategories that emerged after the focus group discussions.

MACRO	MESO	MICRO
Governance	Structure	Clinical learning experience
**Policy and Procedure:** *Academic* (Higher Education Institute) ■ HPCSA guiding document ■ WCPT guidelines ■ DoH policies ■ Accreditation by national ■ (HPCSA) & International regulatory bodies (WCPT) ■ Institution autonomy (even though guidance came from regulatory bodies) *Clinical site* ■ Memorandum of Agreement between the university and clinical site **Resources** Human (mainly) Transport	**Academic structure:** Curriculum Programme evaluation **Operational structure at clinical site:** Clinical supervision Student evaluation	**Positive and negative learning experiences due to:** **Academic and operational structure (imposed on students)** **Clinical educator:** Characteristics (role models) Supervisory style **Student factors:** Characteristics Personal issues Resources Learning style Input: ■ Curriculum ■ Policy and procedure (academic + operational)

HPCSA, Health Professions Council of South Africa; DoH, Department of Health; WCPT, World Confederation of Physiotherapy (now known as World Physiotherapy).

### Phase two

**FIGURE 2:**
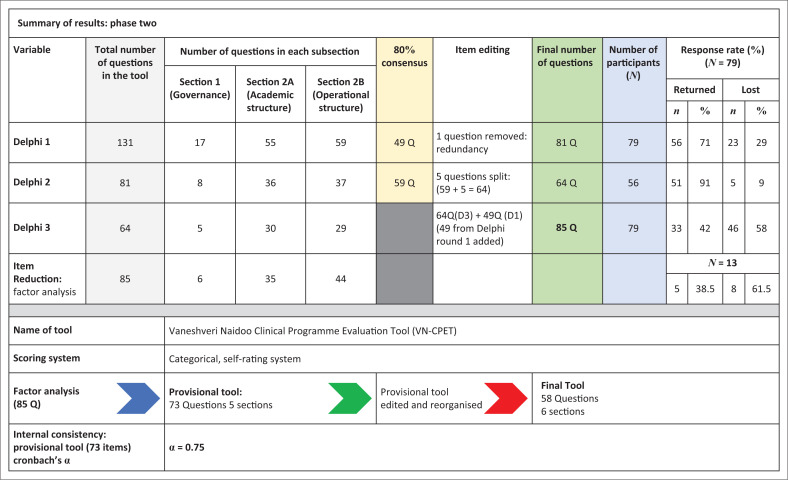
Combined results from phase two of the study.

Figure 2 illustrates the outcomes the Delphi rounds, the exploratory factor analysis and the internal reliability of the tool. The 131 items were reduced to 85 items after the Delphi rounds. Principal component factor analysis reduced the items to 73, and five sections, which were edited and reorganised by the first author to produce the final tool of 58 questions and six sections.

Vaneshveri Naidoo Clinical Programme Evaluation Tool (VN-CPET) was the name chosen for our tool, after numerous suggestions. The VN-CPET is a self-administered programme evaluation tool. Scoring options reflect self-assessment, as illustrated in Table 2.

**TABLE 2:** Scoring system.

Number	Statement	Scoring
3.	Is a comprehensive orientation programme undertaken at the clinical site when students start each clinical rotation?	Always Sometimes Never
4.	Describe your remediation programme (RP), if available?	Comprehensive Needs driven Not done

Five completed questionnaires (38.5%) were used to run principal component factor analysis and determine the internal reliability of the questions. Exploratory factor analysis split the tool into five sections and a total of 73 questions, with an acceptable internal consistency of 0.75 (Bolarinwa 2015; Hulin, Netemeyer & Cudeck 2001).

The authors reviewed the provisional tool following factor analysis and further reduced items due to redundancy, which resulted in the final tool containing 58 questions. We also reorganised the sections of the tool based on my experience as a CC (praxis), which resulted in the final tool containing six domains:

1. Section 1 – Governance (5 questions)
2. Section 2 – Academic processes (5 questions)
3. Learning exposure (6 questions)
4. Clinical orientation (7 questions)
5. Clinical supervision (18 questions)
6. Monitoring and evaluation and quality assurance (19 questions)

See Appendix 1 for the complete tool.

### Phase three

In this phase, although 71% (25) responded (*n* = 35), only 68% (17) of the participants completed the entire questionnaire. Eight (32%) questionnaires were incomplete.

**FIGURE 3:**
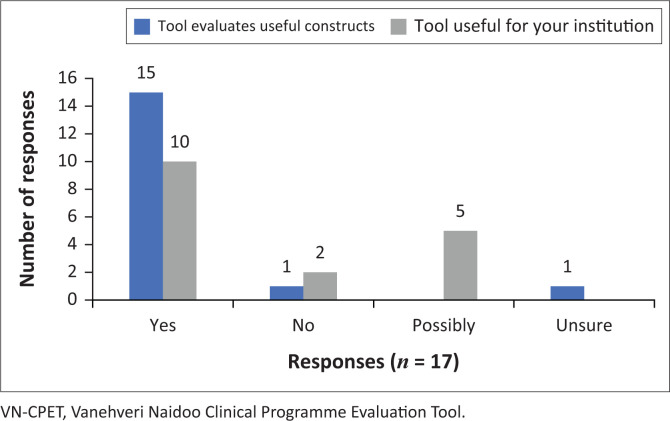
Feasibility and usefulness of clinical programme evaluation tool.

Figure 3 illustrates that 88% of the respondents found that the VN-CPET evaluates useful constructs of clinical education, while 59% indicated that VN-CPET would be useful for their institution. Of the remainder, 29% thought it was likely to be useful for their institution, thus elevating the institutional usefulness of the tool to 88%.

**FIGURE 4:**
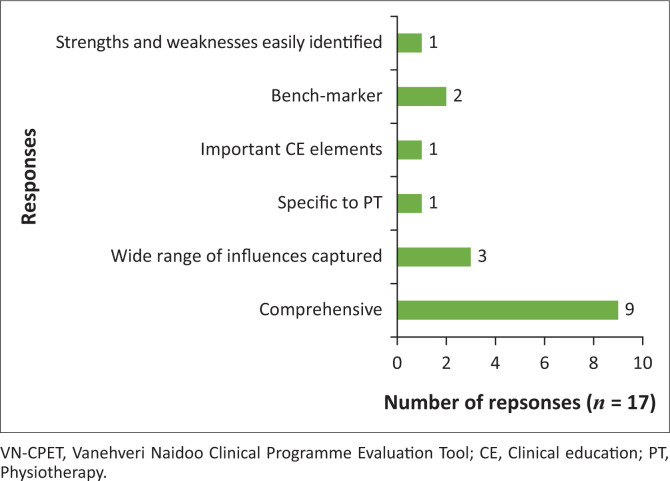
Strengths of clinical programme evaluation tool.

**FIGURE 5:**
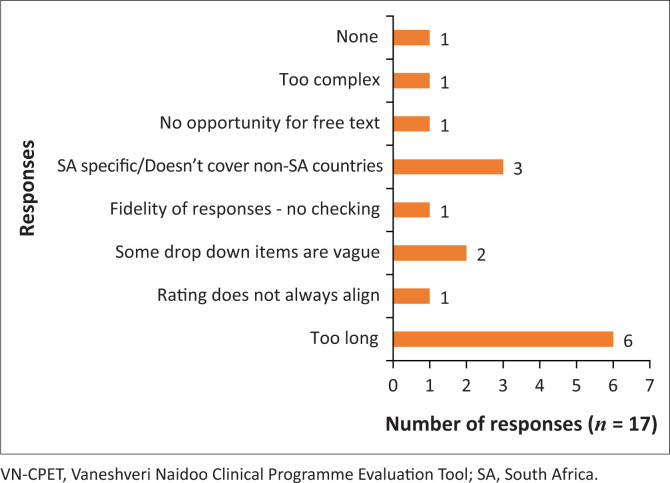
Weaknesses of clinical programme evaluation tool.

In Figure 4, 53% of the respondents commented on the *comprehensiveness* of the tool as a remarkable strength, followed by the *wide range of influences* that were captured (18%); and 12% thought it was a *bench-marker*. The length of the tool was its major drawback, which was pointed out by 35% of the respondents (Figure 5).

## Discussion

A physiotherapy clinical education programme requires independent and objective evaluation to determine its merits and shortcomings. Such an evaluation enables educators/academics to strengthen aspects, add quality assurance mechanisms where required, or remove unintended effects (Frye & Hemmer 2012; Stufflebeam 2003). Mixed methods (Strohschein et al. 2002) enabled us to explore the length, breadth and depth of physiotherapy clinical education by using FGDs, the Delphi method, exploratory factor analysis and a cross-sectional survey. The FGDs, a data-intensive process (Doody, Slevin & Taggart 2013; Greenwood et al. 2017; Portney & Watkins 2009; Winke 2017), immersed us in the complexities of physiotherapy clinical education. Three themes unfolded from the FGDs: *governance (macro), structure (meso) and experience (micro),* which emphasised the complex interaction of these themes. The qualitative leg thus allowed us to gain insight into the complexities of clinical education (Moretti et al. 2011), as affirmed by numerous scholars (Higgs 1993; Jette et al. 2014; McCallum et al. 2013; Patton, Higgs & Smith 2018; Stachura et al. 2000; Strohschein et al. 2002). Additionally, the impact on students’ clinical learning, as a result of the structure and processes of a clinical education programme, was discussed.

The preliminary tool of 131 items was refined into the provisional tool (73 questions; five sections) by using two key processes: the Delphi process and exploratory factor analysis. The Delphi process, through *a posteriori* consensus (knowledge based on experience or personal observation), which was set at 80% (Maleka et al. 2017), was used to determine the items and domains of the tool. This was appropriate in our study, as we were able to cost-effectively include several participants in a field of study where there is a paucity of information (Diamond et al. 2014; Okoli & Pawlowski 2004; Powell 2003; Trevelyan & Robinson 2015). Exploratory factor analysis further delineated the items and domains of the tool by grouping similar variables into smaller groups, while eliminating variables that had a low factor loading and/or lowered the internal consistency of the items. (Portney & Watkins 2009). The final tool, through praxis, was created: 58 questions and six sections.

An acceptable internal reliability of 0.75 (*n* = 73) indicated that that the inter-relatedness of the items is satisfactory, and thus the tool will always, consistently measure the diverse, complex and multidimensional construct of physiotherapy clinical education. A high internal consistency (> 0.90) does not always mean the tool is more reliable; it could indicate that there is a high degree of redundancy in the items (McCrae et al. 2011; Taber 2018; Tavakol & Dennick 2011). An acceptable internal consistency appears to be more suitable for a newly developed tool (Bolarinwa 2015; Taber 2018; Tavakol & Dennick 2011).

Weiner et al. (2017) confirmed that the acceptability, appropriateness, and feasibility of an instrument must be determined to ensure its implementation. In other words, is this innovation satisfactory, fit for purpose, and useable in its context? A decisive ‘yes’ by 88% (15 out of 17; *n* = 17) of the participants was the reply. The VN-CPET was deemed comprehensive and a bench-marker that captured a wide range of influences, although it is long. This tool, therefore, considers everything that is important in evaluating the effectiveness and quality of a physiotherapy clinical education programme, under these sections: *governance; academic processes; learning exposure; clinical orientation; clinical supervision; monitoring* and *evaluation,* and *quality assurance.*

*Governance* refers to a multitude of factors: people, roles, structures, and policies. It is a framework under which stakeholders perform activities within regulated boundaries (Bigdeli et al. 2020; Pyone et al. 2017). In this section of the tool, governance refers to policies and agreements that guide the educational programme at a macro, meso- and micro level. Programme governance establishes processes and provides a structure for communication, implementation, and monitoring. It also ensures that policies and best practices are followed. Additionally, it ensures that the programme’s goals and objectives are aligned with the larger institutional and regulatory bodies.

*Academic processes* refers to the educational strategies (curriculum; teaching; learning; assessment; resources – human and others) that have been instituted to ensure adequate pre-clinical preparation, while *learning exposure* refers to the hands-on learning opportunities that students experience to ensure competency in all areas of physiotherapy, meeting the needs of their country inclusively, efficiently and cost-effectively (Hirsh et al. 2007). *Clinical orientation* programmes are aimed at enhancing students’ transition (vertically or horizontally) and student success, as they adapt to a new environment (Nguyen et al. 2018; Perrine & Spain 2008).

*Clinical supervision* is central to the effective training of health science students (Delany & Bragge 2009; Ernstzen & Bitzer 2012; Ernstzen, Bitzer & Grimmer-Somers 2009, 2010; Kilminster et al. 2007; Laitinen-Väänänen, Talvitie & Luukka 2007; Meyer, Louw & Ernstzen 2019; Patton et al. 2018; Pront, Gillham & Schuwirth 2016). It is integral to teaching and learning, and achieving competency in health science education (Laitinen-Väänänen et al. 2007; McAllister, Higgs & Smith 2008)

*Monitoring and evaluation* and *quality assurance* are different processes that occur simultaneously to ensure that objectives and goals are met; to identify and mitigate unintended effects; to determine the effectiveness and impact of an activity or programme, and to ensure that delivery of activities and their outcomes match the gold standard (Annecke 2008; Jette et al. 2014; Myezwa, M’Kumbuzi & Mhuri 2001; Stachura et al. 2000; Tsinidou, Gerogiannis & Fitsilis 2010).

Monitoring is the continual assessment of a project or programme to determine its intended and unintended effects (formative evaluation), whereas evaluation is the periodic retrospective assessment of a project or programme to determine its worth: relevance, impact, effectiveness, efficiency and sustainability (summative evaluation) (Annecke 2008; Porter & Goldman 2013; Stem et al. 2005; Stone-Jovicich et al. 2019). Quality assurance, on the other hand, is the evaluation of activities against a gold standard or guideline (Stachura et al. 2000). Summative and formative evaluation of physiotherapy clinical education informs the evaluator of the length, breadth, and depth of physiotherapy clinical education. The VN-CPET enables the aforementioned and allows quality assurance measures to be inserted where necessary. Most importantly, the VN-CPET provides a standardised, valid and reliable way of evaluating a physiotherapy clinical education programme.

## Conclusion

The VN-CPET reflects the complexity and diversity of clinical education, due to its ability to be ‘comprehensive’ and to capture a ‘wide range of influences’. Although long, it was found to be acceptable, appropriate, and feasible. Furthermore, the VN-CPET is a valid and reliable tool and can be used to objectively evaluate the effectiveness and quality of a physiotherapy clinical education programme. Even though the scoring system is subjective, an evaluative response is obtained. A link to the online tool can be requested from the corresponding author.

The strength of the VN-CPET lies in its rigorous development using mixed methods (Strohschein et al. 2002), and the South African context is by no means a barrier to its global application in clinical physiotherapy education: the educational framework of physiotherapy clinical education is the same, despite different contexts. This tool will be shortened, and the scoring system refined in future studies.

The limitations include the subjectivity in the existing scoring system; the length of the tool, which is a potential barrier to its use; and the purposive sampling that was used to determine the feasibility and usefulness of this tool. Therefore, this tool should be used bearing these limitations in mind.

## Appendix 1: The Vaneshveri Naidoo Clinical Programme Evaluation Tool (VN-CPET)

The final VN-CPET tool of 58 items and six subsections was developed: ‘Governance; Academic Processes; Learning Exposure; Clinical Orientation; Clinical Supervision and Monitoring & Evaluation and Quality Assurance’.

### How to use this tool

The assessor using this tool answers the question in column two first. After answering the question, the assessor then self-evaluates their answer by choosing an option in column three under scoring. For example, question 1, if your answer was only *University policies*, then the scoring option chosen would be ‘some’; alternatively, if all three options were chosen (macro, meso and micro), then the scoring option you would choose is ‘All’. The principle is answer question is column two first, and then you score your answer. The scoring option provided appraises the answer of the assessor (it’s a self-evaluation system)

**TABLE 1-A1:** The Vaneshveri Naidoo Clinical Programme Evaluation Tool (VN-CPET).

Number	Section	Scoring
**Section 1: Governance**		
1.	Which of the following policies inform your physiotherapy clinical education programme? *Macro level: DHET, DoH (HPCSA, Act 54 of 1974*) Meso level: *University policies* *Micro level:* Departmental policies	2 All 1 Some 0 None
2.	Do you comply with the HPCSA guidelines when structuring your physiotherapy clinical education programme?	2 Always 1 Sometimes 0 Never
3.	Does institution autonomy supersede the guiding documents from the HPCSA?	0 Always 1 Sometimes 2 Never
4	Does an active Memorandum of Agreement exist between the university or your department and the clinical training sites?	2 Yes 1 Working towards 0 Not achieved
5.	How are challenging operational issues pertaining to student training resolved at the clinical placements?	2 Appropriate 1 Somewhat appropriate 0 Not appropriate
6.	Are students aware of the conflict resolution process that must be followed?	2 Always 1 Sometimes 0 Never
**Section 2: Academic processes**		
7.	Are the theory components completed prior to the clinical exposure?	2 Always 1 Sometimes 0 Never
8.	Are there clear aims and objectives (AO) specified for each clinical exposure?	2 Always 1 Sometimes 0 Never
9.	What opportunities are available for students to learn different languages?	2 Always 1 Sometimes 0 Never
10.	Describe your remediation programme, if available?	2 Comprehensive 1 Needs driven 0 Not done
11.	Describe your clinical supervisor training programme	2 Comprehensive 1 Needs driven 0 Not done
**Section 3: Learning exposure**		
12.	Does the clinical exposure reflect the national/provincial burden of disease?	2 Always 1 Sometimes 0 Never
13.	How diverse is your clinical exposure regarding the different levels of health care: Primary, Secondary, Tertiary level; Private Health Care?	3 Maximally diverse 2 Moderately diverse 1 Minimally diverse
14.	Describe the clinical exposure that students experience in your institution. (clinical exposure refers to clinical experience in these areas: cardiopulmonary, ICU, orthopaedics: in-patients & out-patients/sport; neurology; paediatrics, community/public health etc.)	3 Wide 2 Fair 1 Narrow
15.	How many clinical areas are the students exposed to in their years of undertaking clinical education as an examination subject? (list each year separately, and then add to obtain the total).	3 > 6 areas 2 4–6 areas 1 1–3 areas
16.	How many hours per week do students spend training clinically in their years of undertaking clinical education as an examination subject? (List year separately and then add to obtain the total)	3 31 h – 40 h 2 21 h – 30 h 1 1 h – 20 h
17.	Describe the nature of the clinical experience/exposure (longitudinal/integrated/silo)	3 Longitudinal 2 Integrated 1 Silo 0 Other
**Section 4: Clinical orientation**		
18.	Do students write a pre-block test prior to starting clinical training?	2 Always 1 Sometimes 0 Never
19.	Are policies and procedures of a clinical placement/site overtly communicated to students on the first day at the placement?	2 Always 1 Sometimes 0 Never
20.	Is a comprehensive orientation programme undertaken at the clinical site when students start each clinical rotation/block?	2 Always 1 Sometimes 0 Never
21.	Are students orientated to all aspects and areas in physiotherapy department (equipment; forms; bathroom; student room; lockers etc) in which they are placed?	2 Always 1 Sometimes 0 Never
22.	Are students orientated to all necessary areas (layout/architecture) of clinical placement?	2 Always 1 Sometimes 0 Never
23.	Are students introduced to all staff members in the physiotherapy department when starting at a clinical placement?	2 Always 1 Sometimes 0 Never
24.	Are students introduced to the interprofessional team at the clinical site when starting a clinical rotation/block?	2 Always 1 Sometimes 0 Never
**Section 5: Clinical supervision**		
25.	Is a standardised supervision structure applied across all clinical sites during the clinical training?	2 Always 1 Sometimes 0 Never
26.	Is the supervision session patient-centred or student-centred?	2 Patient-centred 1 Student-centred 0 Not considered
27.	State the method of supervision used? (group; one-to-one; peer-led; teacher-led etc)	2 Various 1 One only 0 None
28.	Describe the teaching methods undertaken during clinical supervision sessions. (refer to definitions page)	2 Various 1 One only 0 Not considered
29.	When is feedback generally given during the supervision process?	1 Immediately 2 End of rotation 3 Not given
30.	What method of feedback is used?	1 Verbal 2 Written 3 Peer 4 None 5 Other: specify
31.	How is student autonomy fostered by the clinical educator?	2 Scaffolded approach 1 Abrupt/sudden 0 No autonomy
32.	How is student inter-professional collaboration ensured or encouraged at the clinical site?	2 Appropriate 1 Somewhat appropriate 0 Not done
33.	Are basic clinical skills revised during clinical supervision?	2 Always 1 Sometimes 0 Never
34.	How is students’ patient management facilitated at the clinical site?	2 Appropriate 1 Somewhat appropriate 0 Not done
35.	How are students guided with reading and recording in patients’ files?	2 Appropriate 1 Somewhat appropriate 0 Not done
36.	Do students receive dedicated supervision time from the clinician?	2 Always 1 Sometimes 0 Never
37.	Do students receive dedicated supervision time from the university clinical educator?	2 Always 1 Sometimes 0 Never
38.	Are the factors that affect learning considered by the clinical educator during clinical supervision? (student’s characteristics; learning style; resources and personal issues/emotions: fear, anxiety etc)	2 Always 1 Sometimes 0 Never
39.	Are the expectations of the supervisor realistic based on the level (year of study) of the student?	2 Always 1 Sometimes 0 Never
40.	What is the student/supervisor ratio? (e.g. 1 supervisor:8 students)	2 Appropriate 1 Somewhat appropriate 0 Not appropriate
**Section 6: Monitoring & evaluation and quality assurance**		
41.	How do you evaluate your physiotherapy clinical education programme?	2 Evaluated 1 Working towards 0 Not evaluated
42.	Has your clinical education programme been accredited by any professional body/organisation? For example, WCPT; HPCSA	2 Fully accredited (local + international) 1 Partially accredited (local only) 0 Not accredited
43.	List the quality assurance measures (QAM) you have in place for your physiotherapy clinical education programme?	2 Rigorous QAM 1 Working towards QAM 0 No QAM
44.	What resources do you have that strengthen your clinical education programme?	2 Always 1 Sometimes 0 Never
45.	Is there alignment between the curriculum and clinical exposure?	2 Always 1 Sometimes 0 Never
46.	Are your clinical training sites/placements accredited to train students?	2 Yes 1 Working towards 0 No
47.	Who accredits your clinical training sites?	3 University accreditor 2 Regulatory body 1 Professional association 0 Other: specify
48.	What steps are taken to create a positive and safe learning environment at the clinical site?	2 Several 1 Few 0 None
49.	Do students have an opportunity to share their clinical learning experiences with the academic and clinical departments (positive and negative)?	2 Always 1 Sometimes 0 Never
50.	What support structures are in place for your students? (e.g.: mentor/peer support programme/debriefing etc.)	2 Adequate support 1 Some support 0 No support
51.	Which factors influence learning outcomes of students? (positive/negative) (personal/environmental)	2 Several 1 Few 0 None
52.	Which graduate attributes are aimed for in your students? Professional Communicator Collaborator Leader/manager Health Advocate	2 All 1 Some 0 None
53.	What assessment methods are used during clinical practice?	2 Appropriate 1 Somewhat appropriate 0 Not appropriate
54.	How do students travel to clinical sites?	2 Mainly university transport 1 Mainly own transport 0 Mainly public transport
55.	If the clinical site is greater than 60 km away from the university, is free accommodation provided for students at the clinical site or nearby?	2 Always 1 Sometimes 0 Never
56.	Do you have a dedicated clinician at the clinical site that liaises with the university should any operational or student issue arise at the clinical sites?	2 Always 1 Sometimes 0 Never
57.	Do you have a clinical coordinator at the university to coordinate the clinical programme between the university and clinical site/placement?	2 Yes 1 Working towards 0 No
58.	Are clinical educators’ permanent employees of the university?	2 Always 1 Sometimes 0 Never

HPCSA, Health Professions Council of South Africa; DHET, Department of Higher Education and training; DoH, Department of Health; WCPT, World Confederation for Physical Therapy; ICU, Intensive Care Unit; CE, clinical educator; sup, supervisor; st, student; CPET, Clinical Programme Evaluation Tool.

## Appendix 2: Phase 3 participants

VN-CPET was emailed to the Heads of Departments and/or clinical coordinators of each university (i.e. the tool was emailed to the HOD only where departments did not have a clinical coordinator, and to both the HOD and clinical coordinator in departments that had a clinical coordinator, and therefore it was emailed to 35 participants in total).

**TABLE 1-A2:** Phase 3 participants.

Country	University
**Africa:** Ghana Malawi Rwanda Uganda Zambia Zimbabwe South Africa	University of Ghana University of Malawi University of Rwanda Mbarara University of Science and Technology University of Zambia University of Zimbabwe Sefako Makgatho Health Sciences University (SMU) University of Cape Town (UCT) University of Kwazulu-Natal (UKZN) University of Pretoria (UP) University of Stellenbosch (US) University of the Free State (UFS) University of the Western Cape (UWC) University of the Witwatersrand (Wits)
**Australia**	University of Newcastle (UoN) University of Sydney
**Canada**	University of Alberta
**England**	Keele University University of Brighton (UoB) University of Southampton
**Europe**	University of Jyväskylä
**Middle East**	University of Saint-Joseph-Lebanon
**New Zealand**	University of Otago
**Singapore**	National University of Singapore
**USA**	University of Stockton New Jersey University of Florida
**South America**	University of São Paulo

VN-CPET, Vaneshveri Naidoo Clinical Programme Evaluation Tool; HOD, heads of departments; USA, United States of America.
